# Replacing vertical actions by mouse movements: a web-suited paradigm for investigating vertical spatial associations

**DOI:** 10.1007/s00426-022-01650-6

**Published:** 2022-02-07

**Authors:** Emanuel Schütt, Ian Grant Mackenzie, Barbara Kaup, Carolin Dudschig

**Affiliations:** 1 Department of Psychology, Language and Cognition Research Group, University of Tübingen, Schleichstr. 4, 72076 Tübingen, Germany; 2 Department of Psychology, Biological Psychology Research Group, University of Tübingen, Schleichstr. 4, 72076 Tübingen, Germany

## Abstract

The number of web-based studies in experimental psychology has been growing tremendously throughout the last few years. However, a straightforward web-based implementation does not exist for all types of experimental paradigms. In the current paper, we focus on how vertical response movements—which play a crucial role in spatial cognition and language research—can be translated into a web-based setup. Specifically, we introduce a web-suited counterpart of the vertical Stroop task (e.g., Fox & Shor, in Bull Psychon Soc 7:187–189, 1976; Lachmair et al., in Psychon Bull Rev 18:1180–1188, 2011; Thornton et al., in J Exp Psychol Hum Percept Perform 39:964–973, 2013). We employed nouns referring to entities typically located in lower or upper vertical space (e.g., “worm” and “bird”, respectively) in Experiments 1 and 2, and emotional valence words associated with a crouched or an upward bodily posture (e.g., “sadness” and “excitement”, respectively) in Experiment 3. Depending on the font color, our participants used their mouse to drag the words to the lower or upper screen location. Across all experiments, we consistently observed congruency effects analogous to those obtained with the lab paradigm using actual vertical arm movements. Consequently, we conclude that our web-suited paradigm establishes a reliable approach to examining vertical spatial associations.

## Introduction

Within the last decade, the Internet has become increasingly relevant for behavioral research. This development is currently being boosted by the coronavirus pandemic. Experimental psychologists started to employ the Internet as a research tool in the middle of the 1990s (Krantz & Dalal, 2000; Reips, 2000, 2002a). Up to the present day, the number of studies using the Internet for delivering surveys and running experiments has grown tremendously (Gosling & Mason, 2015; Stewart et al., 2017; Woods et al., 2015). Nevertheless, the potential of the Internet with respect to conducting behavioral research—especially in terms of chronometric studies—is not yet entirely realized. In the present article, we focused on providing a setup that replaces vertical response movements by means of mouse movements on the horizontal plane inducing vertical stimulus movements on the computer screen. Importantly, this setup is easy to implement, allows web-based data collection, and can also simplify research in the lab.

Web-based data collection provides the opportunity to overcome several issues associated with classical lab research. Particularly, the Internet and online labor markets (e.g., Amazon Mechanical Turk or Prolific) enable researchers (1) to gather data of participants with a much more diverse background than the typically recruited university students (for issues with “WEIRD” samples in behavioral science see Henrich et al., 2010), (2) to sample data in short periods of time, and (3) to recruit participants with special characteristics (Birnbaum, 2004; Reips, 2000; Stewart et al., 2017; Woods et al., 2015). Naturally, we also face challenges and disadvantages when we decide to deliver surveys or conduct experiments via the Internet. Especially, researchers must deal with the fact of losing experimental control (e.g., Duffy, 2002; Gosling & Mason, 2015; Nosek et al., 2002). For example, the experimenter has reduced options for making sure that the participants follow the instructions and take their participation seriously. Due to the lack of personal interaction, there is no way of clearing up misunderstandings or answering questions (Reips, 2002b). Moreover, several authors mention problems with respect to data security and research ethics, such as the issue that the experimenter cannot guarantee that the participants read and comprehended the informed consent statement (e.g., Buchanan & Williams, 2010; Emery, 2014; Kraut et al., 2004; Rhodes et al., 2003). In addition, lab paradigms and their web-based counterparts will rarely be fully identical (for a similar issue regarding psychological tests see Buchanan, 2002). However, there are ways and means of mitigating, managing, and (partially) solving the challenges and disadvantages that are typically related to conducting psychological research via the Internet (see, for example, Aust et al., 2013; Reips, 2000, 2002a, 2002b, 2009).

Before turning to the paradigm introduced and evaluated in the present research, we will briefly discuss the issue of timing in web-based reaction time (RT) experiments. Certainly, there exist some reservations mainly arising from arguments such as software and technology constraints and the increasing situational and technical variance in web-based data collection (Hilbig, 2016). However, it seems that the respective issues are significantly less severe than originally expected. For instance, Reimers and Stewart (2015) systematically investigated the accuracy of RT measurements in web-based experiments across different computers, operating systems, ways of implementation (Adobe Flash vs. HTML5), and browsers. Their results showed that RT effects can be detected accurately in most setups. Importantly, for a between-subjects design with two conditions, they demonstrated that the noise generated by hardware and software variability can easily be compensated by slightly raising the sample size. Moreover, for a within-subjects design with two conditions, they detected virtually no disadvantages (see Neath et al., 2011, for results with Apple computers). In a recent review, Stewart et al. (2017) stated that “it is now possible to measure reaction times sufficiently accurately in web experiments using HTML5 and Javascript” (p. 739). Additionally, several (classical) RT paradigms have been tested and validated in online settings, mostly either by replicating the results of the original lab studies or by administering the paradigm in a lab setting and in an online setting. For instance, lab and online results were comparable for the Stroop effect, the Simon effect, the flanker task, the attentional blink task, task-switching costs, visual cuing, visual search, the word frequency effect, the right-visual-field advantage for word recognition, and syntactic priming in sentence production (Corley & Scheepers, 2002; Crump et al., 2013; Hilbig, 2016; Linnman et al., 2006; McGraw et al., 2000; Semmelmann & Weigelt, 2017; Simcox & Fiez, 2014).

In general, web-based experimenting seems to be completely feasible and acceptable with respect to experimental designs that require keypresses on a standard keyboard. In addition, in recent years, mouse-based paradigms have been successfully used across various research domains to gain insights into cognitive processes (for reviews, see Freeman, 2018; Schoemann et al., 2021; Stillman et al., 2018). For instance, researchers employed mouse-tracking to examine semantic categorization (Dale et al., 2007), to study social categorization (Freeman et al., 2010), to detect response difficulty in online surveys (Horwitz et al., 2017), and to establish a potential early marker of mild cognitive decline (Seelye et al., 2015). In the present article, we aimed at making use of these developments for introducing a web-suited counterpart to vertical response movements typically recorded in lab-based settings. This sort of vertical response mode plays a crucial role in various branches of behavioral research, such as spatial cognition, numerical cognition, social cognition, and cognitive control (e.g., Dudschig & Kaup, 2020; Ito & Hatta, 2004; Koch et al., 2011; Schneider, 2020; Schubert, 2005; Schwarz & Keus, 2004).

**Fig. 1:**
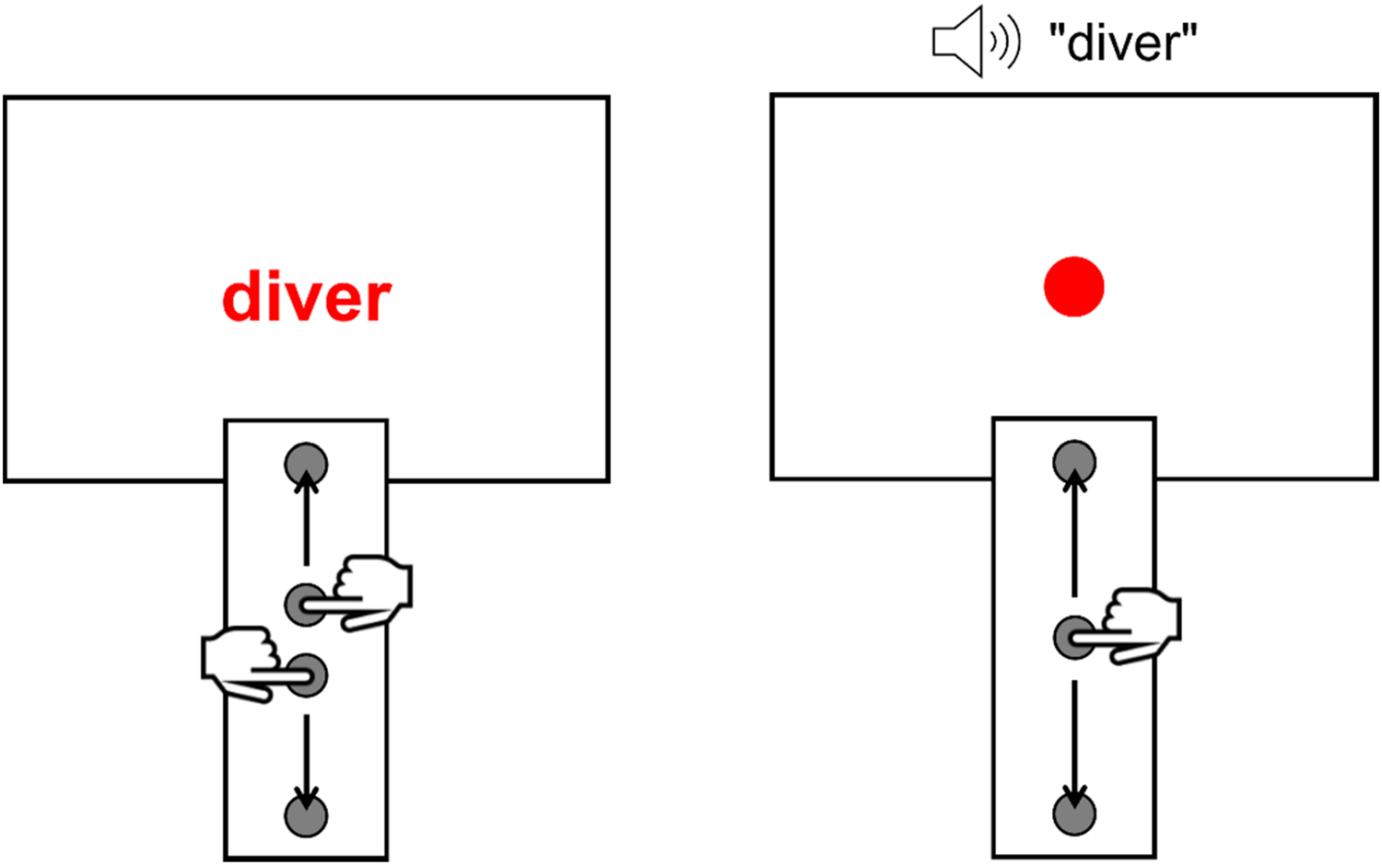
Examples of setups used in research on vertical language-space associations. The illustration on the left shows the experimental setup used by Lachmair et al. (2011, Experiment 2). Participants performed a downward or an upward arm movement to respond to the font color of nouns. The vertically mounted response device had four keys: two middle keys, a lower response key, and an upper response key. Participants initiated a trial by holding both middle keys. A downward (an upward) arm movement included releasing the corresponding middle key, pressing the lower (the upper) response key, and returning to the middle key. The non-responding hand remained on the middle key. The illustration on the right shows the modified setup used by Vogt et al. (2019). In this case, participants performed downward and upward movements with a single arm. Subsequent to the auditory presentation of the language stimulus, they responded to the color of a circle displayed at the center of the screen

We based our work on research using the vertical Stroop task. For example, Lachmair et al. (2011) employed such a setup (see also Dudschig & Kaup, 2017; Thornton et al., 2013) for verifying assumptions that were derived from the experiential-simulations view of language comprehension (Barsalou, 1999; Glenberg & Kaschak, 2002; Zwaan & Madden, 2005). In their vertical Stroop task, Lachmair et al. (2011, Experiment 2) presented participants with nouns referring to entities that are typically located in lower or upper vertical space (e.g., “worm” and “bird”, respectively). Critically, each noun was displayed in one of four font colors. Depending on the font color, the participants performed a downward or upward arm movement employing a vertical response device mounted in front of them (see Fig. 1). Concretely, they pressed down the two middle keys of the device using their right and left hand to initiate a trial. To respond to the font color of the noun, they released the relevant middle key, pressed the corresponding lower or upper response key and returned to the released middle key. Importantly, the time period from the appearance of the noun until releasing one of the middle keys (releasing time) served as the dependent variable. In line with the original Stroop task (Stroop, 1935) correct responses solely demanded processing the font color. Thus, the task did not require participants to process the nouns. Nevertheless, Lachmair et al. observed a congruency effect of response direction and referent location, indicating that spatial features are indeed activated in a rather automatic manner when people encounter nouns that are associated with a typical vertical location (see Thornton et al., 2013, for converging results). Remarkably, the results suggest that the setup is able to detect spatial congruency effects that are likely smaller than those obtained with the standard spatial Stroop task using the words “up” and “down” (Fox & Shor, 1976; Fox et al., 1971). Hence, establishing a reliable web-based counterpart to this type of setup seems to be highly valuable. Moreover, in recent years, setups with a vertical response dimension have become increasingly relevant in the various fields of behavioral research (e.g., spatial cognition; language comprehension; numerical cognition; social cognition) that have an interest in spatial associations (e.g., Ahlberg et al., 2018; Dudschig et al., 2012, 2014, 2015; Gevers et al., 2006; Günther et al., 2018, 2020; Hill & Lapsley, 2009; Ito & Hatta, 2004; Meier et al., 2007; Öttl et al., 2017; Schubert, 2005; Vogt et al., 2019; Zanolie et al., 2012; Zhai et al., 2018).

In our web-suited counterpart to the manual vertical Stroop paradigm, participants used their computer mouse to respond to the font color of words: They dragged each word either to the lower or to the upper screen location. Consequently, spatial congruency was operationalized via the correspondence of spatial information associated with the language stimulus (e.g., “grave” vs. “satellite”) and the mouse movement that was directly coupled with visually sensible action effects (i.e., perceiving the word moving downwards or upwards, respectively). Noteworthy, in a series of five experiments (Schütt et al., 2021), we recently tested a similar paradigm. In this paradigm, our participants responded to the font color of nouns by performing stationary keypresses. Importantly, pressing a response key consistently produced an immediate visual feedback at the lower or upper end of the screen. Interestingly, we did not reliably observe congruency effects, indicating that visual feedback following simple keypress responses does not suffice to induce the sort of congruency effects studied in research domains that typically demand vertical responses. However, we hypothesized that coupling visual action effects with a more distinct involvement and activation of the motor system—as realized by introducing mouse movements—might be more similar to previous lab-based approaches.^1^ Additionally, using mouse movements seems to be a rather convenient solution: The computer mouse is one of the standard input devices available for web-based research and allows implementing experimental procedures that involve the motor system in a more prominent way.

In three mouse movement experiments we explored whether our web-suited paradigm is appropriate for studying language-space associations in a reliable manner. We started by running web-based replications of the study by Lachmair et al. (2011, Experiment 2) that investigated the automatic activation of spatial information during processing implicit location words, once with native English speakers (Experiment 1) and once with German native speakers (Experiment 2). In Experiment 3, we applied the web-suited paradigm to another group of words, namely valence words referring to emotions that are typically associated with a slouched or an upright bodily posture (i.e., with spatial experiences that are characterized by having a down vs. an up component; see Dudschig et al., 2015; Meier & Robinson, 2004). Importantly, using the lab-based vertical Stroop paradigm of Lachmair et al., it has already been shown that these words influence subsequent vertical arm movements in accordance with the spatial experiences they are related to (see Dudschig et al., 2015, Experiment 3). Therefore, the third experiment enables us to assess the generalizability of our web-suited paradigm by means of testing it with respect to yet another well documented lab-based finding.

## Experiment 1

### Method

#### Transparency and openness

For all experiments, we report our procedure to determine the exact sample size. All data sets and the original and an updated version of the jsPsych plugin for implementing the mouse movement task are publicly available at https://doi.org/10.5281/zenodo.4557024. We will upload analysis scripts at the same place upon publication. All data exclusions, software employed for statistical analyses, and study materials are mentioned and described in the Method sections at an appropriate place. The current research was not preregistered. Ethics approval for the study was obtained from the Ethics Committee for Psychological Research at the University of Tübingen (Identifier: 2018_0831_132).

#### Participants

We based the sample size of the experiment on the lab-based work that served as the starting point for the current research (i.e., Lachmair et al., 2011, Experiment 2 [*N* = 24]). First, we conducted a power analysis using MorePower (Version 6.0.4; Campbell & Thompson, 2012). For this purpose, we referred to the results Lachmair et al. (2011, Experiment 2) reported for the critical congruency effect of response direction and referent location (by-participants analysis of variance; $${\eta }_{\mathrm{p}}^{2}$$ = 0.24). This revealed that 36 participants are needed for having a test power of 0.90 with respect to the congruency effect of response direction and referent location (for similar results obtained with a simulation-based procedure, see Günther et al., 2018). Given that primary studies in behavioral research tend to overestimate effect sizes (Fanelli & Ioannidis, 2013), we then also considered the advice of Simonsohn (2015) that replications should have 2.5 times as many observations as the original study. To incorporate both criteria when setting the sample size, we aimed at collecting the data of 60 participants. In Experiment 1, we recruited native English speakers via Amazon Mechanical Turk. In accordance with Lachmair et al., we discarded participants with a low accuracy rate (less than 90% of correct trials in at least one experimental condition). This procedure resulted in a final sample of 55 participants (i.e., five participants were excluded due to the accuracy rate criterion). The age of the participants (30 males, 25 females; 46 right-handed, eight left-handed, one ambidextrous) ranged from 21 to 65 years (*M* = 38.62 years, *SD* = 10.88 years). The experiment took about 25 min to complete. All participants gave informed consent and received $4.00 in return for participation.

#### Apparatus and stimuli

We created our online experiments by use of jsPsych (Version 6.1.0; de Leeuw, 2015), which is an open-source JavaScript library for generating web-based behavioral experiments. To implement the mouse movement task, we employed a custom-made jsPsych plugin (publicly available at https://doi.org/10.5281/zenodo.4557024). We instructed our participants to use only either a desktop computer or a laptop and a standard computer mouse for conducting the experiments.

A black colored plus sign served as fixation cross. The experimental stimuli were derived from the word list of Lachmair et al. (2011, Experiment 2), which comprised German nouns denoting objects that are typically associated with a lower or an upper vertical location. First, we translated the word list into American English. As a result of the translation, some of the nouns were composed of more than a single word (e.g., “roof beam”; “high wire”; “sole of foot”). However, this was not the case for any of the original German nouns. Therefore, we deleted these nouns. Finally, we randomly excluded further nouns to have a list with the same number of down and up words. This produced a final word list with 32 down words and 32 up words (see Appendix). We examined the nouns with respect to length and frequency. For determining frequency classes, we used an English news corpus that was based on texts from 2016 (available at https://wortschatz.uni-leipzig.de). Down words (*M* = 5.47 letters, *SD* = 1.41 letters) and up words (*M* = 5.53 letters, *SD* = 1.92 letters) did not differ significantly in length, *t*(62) = − 0.15, *p* = 0.883. Likewise, there was no significant difference in terms of frequency, *t*(61) = 0.67, *p* = 0.504 (down words: *M* = 12.45, *SD* = 2.10; up words: *M* = 12.06, *SD* = 2.49).^2^ Sixteen additional nouns (e.g., “book”; “letter”; “machine”) served as stimuli in the training session. These nouns were not associated with any typical vertical location (see Lachmair et al., 2011, Experiment 3). The stimuli were presented in blue (RGB: 0, 0, 255), orange (RGB: 255, 165, 0), green (RGB: 0, 128, 0), and red (RGB: 255, 0, 0) font color on a white background. In the training session as well as in the experimental session, we gave feedback by displaying either “Correct!” (if the response was correct) or “Wrong!” (if the response was wrong). The feedback was shown in black letters.

#### Procedure

We instructed our participants to run the experiment in an interference-free environment. The experiment consisted of a training session and an experimental session. During the training session, participants should get familiar with the task. We presented each training stimulus once in one of the four font colors. Thus, the training session comprised 16 trials. All font colors appeared equally often. The order of trials was randomized. After the training session, a self-paced break followed. This break included performance feedback (i.e., information on the percentage of correct responses and the average response time) and a reminder with respect to the experimental task.

Participants then proceeded with the experimental session, which was composed of four blocks. In each block, we displayed each of the down words and each of the up words once in one of the four font colors. Hence, an experimental block contained 64 trials. We presented each noun in a different font color in each block. Since there were four blocks and four different font colors, all nouns appeared exactly once in each font color during the experimental session. Furthermore, we balanced the font colors within the blocks (i.e., all font colors were used equally often per block). The order of the trials and the order of the blocks were randomized. After each block, there was a self-paced break. Again, this break included a performance feedback and a reminder regarding the experimental task.

**Fig. 2:**
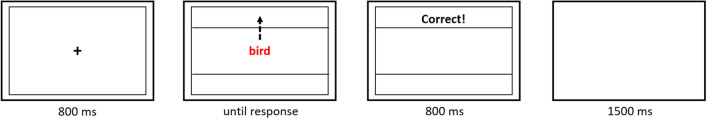
Procedure of a correctly answered trial in Experiment 1. Each trial started with the presentation of a fixation cross. Concurrently, a frame appeared that marked the experimentally relevant part of the screen. Then, a lower and an upper target area was introduced by displaying a borderline at the lower and at the upper end of the framed part of the screen. Moreover, the stimulus replaced the fixation cross. The participants used their mouse to respond to the font color of the stimulus: They dragged the stimulus either to the lower or to the upper target area. Once the stimulus was completely located in one of the target areas, it was replaced by the feedback. Finally, the intertrial interval followed before the next trial started

Figure 2 illustrates the trial procedure. Each trial started with showing simultaneously (1) a frame that marked the experimentally relevant part of the screen (90% of the available display height and 90% of the available display width) and (2) the fixation cross (800 ms). Subsequently, a lower and an upper target area was introduced by displaying a borderline at the lower and at the upper end of the framed part of the screen. At the same time, the stimulus replaced the fixation cross. The task was to react to the font color of the stimulus as fast and as accurately as possible. For this purpose, the participants clicked on the stimulus and dragged it either to the lower or to the upper target area using their mouse. We always mapped two font colors to one response direction (e.g., blue and orange to downward dragging and green and red to upward dragging). The combination of colors to color pairs (blue-orange and green–red; blue-green and orange-red; blue-red and orange-green) and the mapping of color pairs to response directions (i.e., which of the two color pairs was assigned to which of the two response directions) was balanced between participants, resulting in six experimental versions. Once the stimulus was completely located in one of the target areas, it was replaced by the correctness feedback (800 ms). Finally, the intertrial interval (1500 ms) followed before the next trial started.

#### Design and data analysis

The experiment had a 2 $\times$ 2 within-subjects design, with the factors referent location (down vs. up) and response direction (down vs. up). As dependent variables we used measures that likely match the dependent variables considered in the context of the lab-based vertical Stroop task we referred to (see, for example, Dudschig et al., 2015; Lachmair et al., 2011; Thornton et al., 2013). This particularly included a marker reflecting the time period until initiating a response to the presented stimulus (i.e., response selection and planning). Thus, the time period from the appearance of the noun on the screen until the initial movement of the noun (response time) served as a dependent variable. More concretely, the noun needed to be selected via mouse button press, followed by the mouse movement being started (there was no need for moving a certain distance).

We prepared and analyzed response times using the free statistical software R (Version 3.6.2). First, we removed training trials, trials with more than a single mouse click, and incorrectly answered trials. Then, we excluded trials with response times shorter than 100 or longer than 3000 ms. In line with Lachmair et al. (2011), we subsequently applied the two-step procedure suggested by Kaup et al. (2006) to eliminate further outliers. Hence, in a first step, we converted the response times of each participant to *z*-scores. Following this, we discarded response times with a *z*-score that deviated more than two standard deviations from the mean *z*-score of the respective noun in the respective condition. Thus, both differences among the participants and differences among the items were considered. In sum, outlier elimination reduced the data set by less than 7%. We used the R packages lme4 (Version 1.1-21; Bates et al., 2015) and lmerTest (Version 3.1-1; Kuznetsova et al., 2017) to perform a linear mixed effect analysis (see Baayen et al., 2008). Our base model contained fixed effects for referent location and response direction. In contrast to Lachmair et al., we collected data of participants with varying handedness and a rather broad range of age. Consequently, to account for any possible effects of handedness and age, we also added fixed effects for handedness and age to our base model. For determining a suitable random effect structure, we employed the data-driven model selection criterion of Matuschek et al. (2017), which aims at providing a mixed model that balances Type I error rate and power. When performing the procedure, we omitted models that had a singular fit or did not converge. This resulted in incorporating random intercepts for participants and items. For testing our hypothesis (i.e., the interaction of referent location and response direction), we compared our base model to a model that contained an additional fixed effect for the interaction of referent location and response direction by means of a likelihood ratio test.

The time period from the initial movement of the noun until the noun crossed one of the response boundaries (movement time) served as a second dependent variable. We prepared and analyzed movement times in the same way as response times, except that extreme outliers were defined as movement times longer than 1000 ms. Outlier elimination—comprising the two-step procedure recommended by Kaup et al. (2006)—reduced the data set with respect to movement times by less than 6%. The mixed models included random intercepts for participants and items.

Finally, we looked at response correctness. For this purpose, we referred to all correctly and incorrectly answered experimental trials, excluding trials with response times shorter than 100 or longer than 3000 ms (less than 2% of all trials). The analysis followed the procedure used for response and movement times, apart from the fact that we formulated generalized linear mixed models (i.e., mixed effects logistic regressions) to handle the binary outcome variable (response: correct vs. incorrect). These models comprised random intercepts for participants and items.

### Results and discussion

**Fig. 3:**
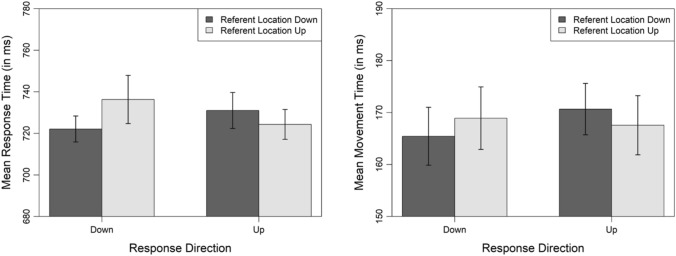
Mean response and mean movement times for correctly answered trials as a function of referent location and response direction in Experiment 1. Error bars denote 95% within-subjects confidence intervals calculated as recommended by Morey (2008

Figure 3 illustrates the mean response and mean movement times as a function of referent location and response direction. For response times, the analysis revealed that the model with an additional fixed effect for the interaction of referent location and response direction explained the data significantly better than the base model, ${\chi }^{2}$(1) = 6.18, *p* = 0.013, effect of referent location: $\beta$ = 10.81, *t* = 1.99, 95% CI [0.15, 21.47], effect of response direction: $\beta$ = 3.15, *t* = 0.64, 95% CI [− 6.45, 12.76], interaction of referent location and response direction: $\beta$ = − 17.24, *t* = − 2.49, 95% CI [− 30.82, − 3.65]. This indicates that responses times were significantly faster when the referent location matched (*M* = 723 ms) compared to mismatched (*M* = 734 ms) the response direction. For movement times, the model with an additional fixed effect for the interaction of referent location and response direction showed a marginally better fit than the base model, ${\chi }^{2}$(1) = 3.58, *p* = 0.058, effect of referent location: $\beta$ = 2.25, *t* = 1.02, 95% CI [− 2.09, 6.58], effect of response direction: $\beta$ = 3.22, *t* = 1.56, 95% CI [− 0.84, 7.28], interaction of referent location and response direction: $\beta$ = − 5.55, *t* = − 1.89, 95% CI [− 11.30, 0.20]. Movements times were slightly faster when the referent location matched (*M* = 167 ms) compared to mismatched (*M* = 170 ms) the movement direction of the noun on the screen. There was no evidence for an interaction of referent location and response direction with respect to response correctness, ${\chi }^{2}$(1) = 0.91, *p* = 0.340, effect of referent location: $\beta$ = − 0.16, *z* = − 0.71, 95% CI [− 0.60, 0.28], effect of response direction: $\beta$ = − 0.07, *z* = − 0.31, 95% CI [− 0.51, 0.37], interaction of referent location and response direction: $\beta$ = 0.30, *z* = 0.95, 95% CI [− 0.32, 0.92]. Error rates were virtually identical when the referent location matched (*M* = 1.20%) compared to mismatched (*M* = 1.31%) the response direction.

We were successful in replicating the congruency effect of referent location and response direction, which provides first support in favor of our paradigm. Particularly, the effect emerged for response times (i.e., the period until initiating the mouse movement). Therefore, the stage of action planning seems to be crucial. Interestingly, this is in line with findings of previous lab-based work on language-space associations (e.g., Dudschig et al., 2015; Günther et al., 2018; Lachmair et al., 2011; Öttl et al., 2017). The effect also clearly tended to be mirrored in movement times. Furthermore, error rates were low, indicating that participants focused on the experimental task.

## Experiment 2

In Experiment 2, we aimed at obtaining further support regarding the validity of our web-suited counterpart to the manual vertical Stroop paradigm. Therefore, we ran a replication of Experiment 1 with German materials and native German speakers.

### Method

#### Participants

We again collected the data of 60 participants. We recruited native German speakers by sending a circular email to the students at the University of Tübingen. The exclusion of participants followed the same accuracy rate criterion as in Experiment 1. This yielded a final sample of 57 participants (i.e., three participants were excluded due to the accuracy rate criterion). The participants (40 females, 17 males; 54 right-handed, two left-handed, one ambidextrous) were between 18 and 36 years old (*M* = 23.67 years, *SD* = 3.51 years). The experiment took about 25 min to complete. All participants gave informed consent. In return for participation, the participants either received partial course credit or participated in a lottery of three vouchers with a value of €60 each.

#### Apparatus and stimuli

In general, apparatus and stimuli were the same as in Experiment 1. This time, however, we conducted the experiment in German. Thus, we presented the original German counterparts of the nouns that we used in Experiment 1 (see Appendix). Again, we examined the nouns with respect to length and frequency. For determining frequency classes, we made use of a German news corpus that was based on texts from 2018 (available at https://wortschatz.uni-leipzig.de). Down words (*M* = 5.88 letters, *SD* = 1.70 letters) and up words (*M* = 5.69 letters, *SD* = 1.42 letters) did not differ significantly in length, *t*(62) = 0.48, *p* = 0.634. Likewise, there was no significant difference in terms of frequency, *t*(62) = 0.57, *p* = 0.571 (down words: *M* = 11.91, *SD* = 1.87; up words: *M* = 11.63, *SD* = 2.08).

#### Procedure

The procedure remained as in Experiment 1.

#### Design and data analysis

Design, data preparation, and data analysis were identical to Experiment 1. Eliminating outliers reduced the data set by less than 5% (response times), less than 6% (movement times), and less than 1% (response correctness). Our method for determining appropriate random effect structures for the mixed models resulted in incorporating random intercepts for participants and items in all models. In addition, the mixed models for analyzing movement times also included by-item random slopes for response location.

### Results and discussion

**Fig. 4:**
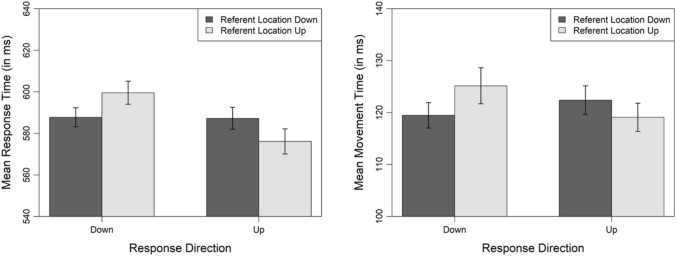
Mean response and mean movement times for correctly answered trials as a function of referent location and response direction in Experiment 2. Error bars denote 95% within-subjects confidence intervals calculated as recommended by Morey (2008)

Figure 4 depicts the mean response and mean movement times as a function of referent location and response direction. For response times, the analysis showed that the model with a further fixed effect for the interaction of referent location and response direction explained the data significantly better than the base model, ${\chi }^{2}$(1) = 27.42, *p* < 0.001, effect of referent location: $\beta$ = 11.34, *t* = 3.63, 95% CI [5.21, 17.47], effect of response direction: $\beta$ = − 0.09, *t* = − 0.03, 95% CI [− 5.84, 5.65], interaction of referent location and response direction: $\beta$ = − 21.67, *t* = − 5.24, 95% CI [− 29.78, − 13.56]. Once again, response times were significantly faster when the referent location matched (*M* = 582 ms) compared to mismatched (*M* = 593 ms) the response direction. For movement times, the model with an additional fixed effect for the interaction of referent location and response direction had a significantly better fit than the base model, ${\chi }^{2}$(1) = 12.51, *p* $<$ 0.001, effect of referent location: $\beta$ = 6.30, *t* = 4.19, 95% CI [3.36, 9.25], effect of response direction: $\beta$ = 3.23, *t* = 1.84, 95% CI [− 0.20, 6.66], interaction of referent location and response direction: $\beta$ = − 9.19, *t* = − 3.72, 95% CI [− 14.04, − 4.34]. Movements times were significantly faster when the referent location matched (*M* = 119 ms) compared to mismatched (*M* = 124 ms) the movement direction of the noun on the screen. Finally, there was an interaction of referent location and response direction regarding response correctness, ${\chi }^{2}$(1) = 21.60, *p* $<$ 0.001, effect of referent location: $\beta$ = − 0.65, *z* = − 3.44, 95% CI [− 1.02, − 0.28], effect of response direction: $\beta$ = − 0.67, *z* = − 3.54, 95% CI [− 1.04, − 0.30], interaction of referent location and response direction: $\beta$ = 1.22, *z* = 4.64, 95% CI [0.71, 1.74]. Errors were rare and the error rate was lower when the referent location matched (*M* = 1.24%) compared to mismatched (*M* = 2.22%) the response direction, ruling out a speed-accuracy tradeoff.

Just as in Experiment 1, we successfully replicated the congruency effect of referent location and response direction. Noteworthy, this time, the effect was reflected in both response times and movement times. In sum, this demonstrates that our web-suited paradigm is suitable for generating spatial congruency effects in a stable manner. Furthermore, by running the experiment in German and recruiting native German speakers, we showed that the paradigm works in different samples and languages.

## Experiment 3

So far, we tested our web-suited counterpart to the manual vertical Stroop task with respect to the stimuli that were used by Lachmair et al. (2011, Experiment 2). Accordingly, we looked at the automatic activation of spatial information during the processing of implicit location words. However, there is strong evidence indicating that language-space associations also exist for various other groups of words. For instance, this applies to power-related words (e.g., “king” and “servant”; Jiang & Henley, 2012; Wu et al., 2016; Zanolie et al., 2012), words describing religious concepts (e.g., “Lord” and “Satan”; Chasteen et al., 2010; Meier et al., 2007), and valence words (e.g., “love” and “danger”; Meier & Robinson, 2004; Santiago et al., 2012). Interestingly, Dudschig et al. (2015, Experiment 3) employed the lab-based vertical Stroop paradigm of Lachmair et al. (2011, Experiment 2) to investigate whether processing a rather specific subset of valence words automatically influences subsequent arm movements. These valence words (e.g., “optimistic” and “disappointed”) were characterized by referring to emotional states that are typically related to an upright or a slouched bodily posture. Crucially, responses in the lab-based experiment were faster when the vertical association of the valence words matched the response direction of the arm movements, reflecting the spatial congruency effect previously reported for implicit location words (see Lachmair et al., 2011, Experiment 2). In Experiment 3, we applied our web-suited paradigm to the posture-specific emotional valence words of Dudschig et al. (2015, Experiment 3). This enabled us to resolve the question whether our web-suited paradigm can replace vertical response movements in the context of further types of spatial associations.

### Method

#### Participants

As in the previous experiments, we based the sample size of the current experiment on the lab-based work that served as a starting point (i.e., Dudschig et al., 2015, Experiment 3 [*N* = 18]). Accordingly, we performed a power analysis using MorePower (Version 6.0.4; Campbell & Thompson, 2012) based on the results Dudschig et al. (2015, Experiment 3) observed for the interaction of response direction and vertical association (by-participants analysis of variance for posture-specific emotional valence words; ${\eta }_{\mathrm{p}}^{2}$ = 0.21). This revealed that 44 participants would be necessary for obtaining a power of 0.90 with respect to replicating the interaction of response direction and vertical association. Once again, we also considered the suggestion of Simonsohn (2015) to determine the number of participants. This resulted in a target sample size of 48 participants.^3^ We recruited native German speakers by sending a circular email to the students at the University of Tübingen. One participant had to be discarded by virtue of the accuracy rate criterion (less than 90% of correct trials in at least one experimental condition). Thus, the final sample consisted of 47 participants. The participants (36 females, 11 males; 42 right-handed, five left-handed) were between 18 and 56 years old (*M* = 22.89 years, *SD* = 5.83 years). The experiment took about 30 min to complete. All participants gave informed consent. In return for participation, the participants either received partial course credit or participated in a lottery of three vouchers with a value of €75 each.

#### Apparatus and stimuli

Apparatus and stimuli remained the same as in Experiment 2, except that we replaced the experimental stimuli. In the current experiment, we showed the list of posture-specific emotional valence words that was used by Dudschig et al. (2015, Experiment 3). The list comprised 20 German valence words referring to pleasant emotions that are associated with an upright bodily posture and 20 German valence words referring to unpleasant emotions that are associated with a rather slouched bodily posture. The full word list can be found in the Appendix.

#### Procedure

Regarding the procedure, there were some minor changes in comparison to Experiment 2. In accordance with Dudschig et al. (2015, Experiment 3), we repeated the experimental stimuli eight times throughout the experiment. Consequently, the experimental session contained eight blocks. In each block, we displayed each of the valence words once in one of the four font colors. Hence, an experimental block comprised 40 trials. All valence words appeared exactly twice in each font color during the experiment. Everything else remained as in Experiment 2.

#### Design and data analysis

The experiment had a 2 $\times$ 2 within-subjects design, with the factors vertical association (down vs. up) and response direction (down vs. up). The dependent variables were defined as in the previous experiments. Data preparation and data analysis were also identical. Outlier removal reduced the data set by less than 5% (response times and movement times) and by less than 1% (response correctness). The method for determining appropriate random effect structures for the mixed models resulted in including random intercepts for participants and items in all models.

### Results and discussion

**Fig. 5:**
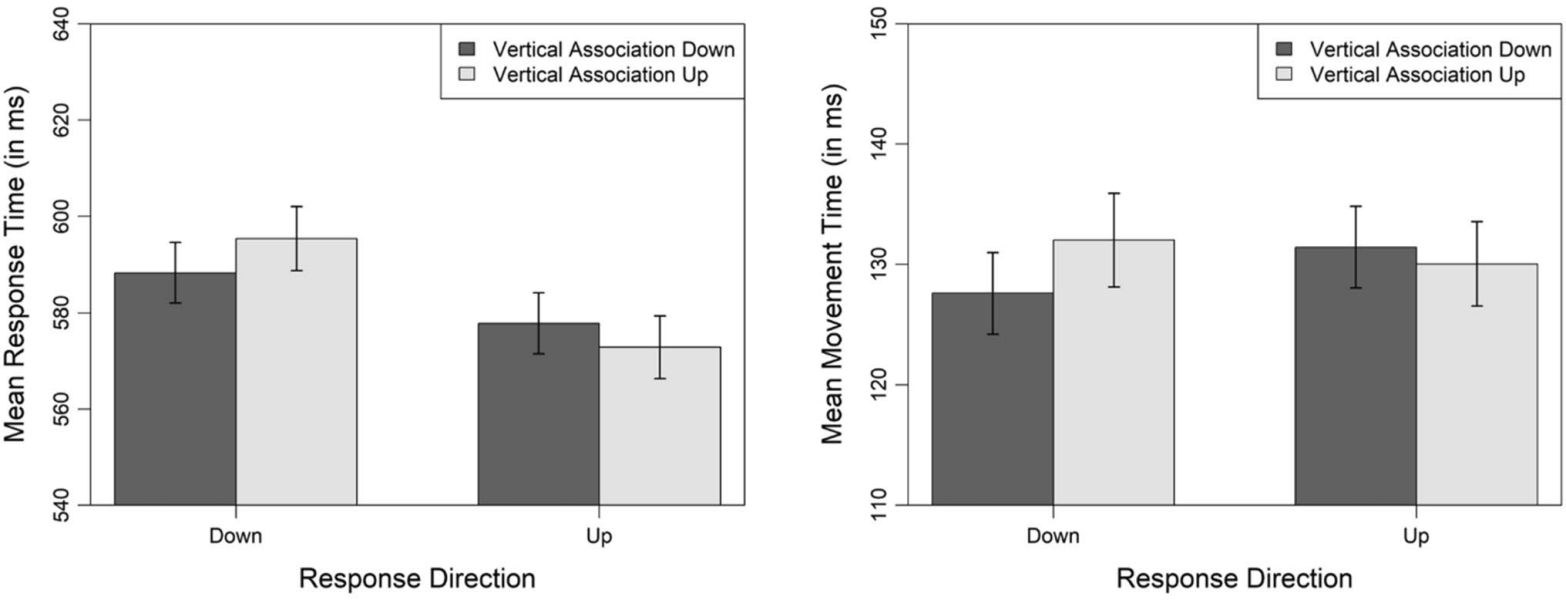
Mean response and mean movement times for correctly answered trials as a function of vertical association and response direction in Experiment 3. Error bars denote 95% within-subjects confidence intervals calculated as recommended by Morey (2008).

Figure 5 displays the mean response and mean movement times as a function of vertical association and response direction. For response times, the analysis demonstrated that the critical model with an additional fixed effect for the interaction of the factors vertical association and response direction explained the data significantly better than the base model, ${\chi }^{2}$(1) = 6.69, *p* = 0.009, effect of vertical association: $\beta$ = 7.19, *t* = 1.97, 95% CI [0.05, 14.32], effect of response direction: $\beta$ = − 8.97, *t* = − 2.79, 95% CI [− 15.29, − 2.66],− interaction of vertical association and response direction: $\beta$ = − 11.79, *t* = − 2.59, 95% CI [− 20.73, − 2.86]. Hence, response times were significantly faster when the vertical association of the word matched (*M* = 581 ms) compared to mismatched (*M* = 587 ms) the response direction. For movement times, the model with a further fixed effect for the interaction of vertical association and response direction had a significantly better fit than the base model, ${\chi }^{2}$(1) = 5.99, *p* $=$ 0.014, effect of vertical association: $\beta$ = 3.94, *t* = 2.59, 95% CI [0.96, 6.92], effect of response direction: $\beta$ = 3.67, *t* = 2.61, 95% CI [0.92, 6.42], interaction of vertical association and response direction: $\beta$ = − 4.86, *t* = − 2.45, 95% CI [− 8.74, − 0.97]. Movement times were significantly faster when the vertical association of the word matched (*M* = 129 ms) compared to mismatched (*M* = 132 ms) the movement direction of the word on the screen. We obtained no evidence for an interaction of vertical association and response direction on response correctness, ${\chi }^{2}$(1) = 1.86, *p* = 0.172, effect of vertical association: $\beta$ = − 0.33, *z* = − 1.60, 95% CI [− 0.73, 0.07], effect of response direction: $\beta$ = − 0.23, *z* = − 1.15, 95% CI [− 0.62, 0.16], interaction of vertical association and response direction: $\beta$ = 0.39, *z* = 1.40, 95% CI [− 0.15, 0.92]. Error rates were similarly low when the vertical association matched (*M* = 1.28%) compared to mismatched (*M* = 1.51%) the response direction.

Crucially, we reproduced the spatial congruency effect with respect to posture-specific emotional valence words. Consequently, the application of our web-suited paradigm is clearly not restricted to solely investigating language-space associations that emerge from implicit location words (see Experiments 1 and 2). Instead, our paradigm seems to establish a reliable suited approach to investigating different varieties of spatial associations.

## General discussion

Throughout the last years, experimental psychologists have increasingly used web-based research tools (Gosling & Mason, 2015; Stewart et al., 2017; Woods et al., 2015). Nevertheless, behavioral science is far from exploiting the full potential of web-based data collection. In the current article, we contributed a paradigm for expanding web-based behavioral research to setups that would typically require vertical response movements. For this purpose, we made use of the vertical Stroop task, which has been particularly relevant to investigating spatial associations across various research fields (e.g., Ahlberg et al., 2018; Dudschig et al., 2012, 2014, 2015; Gevers et al., 2006; Günther et al., 2018, 2020; Ito & Hatta, 2004; Lachmair et al., 2011; Müller & Schwarz, 2007; Öttl et al., 2017; Schwarz & Keus, 2004; Thornton et al., 2013; Vicovaro & Dalmaso, 2021; Vogt et al., 2019).

Interestingly, attempts to replace vertical response movements by providing visual action effects in vertical space failed (Schütt et al., 2021). The present research demonstrates that a more distinct involvement and activation of the motor system is needed. This was realized via coupling mouse movements in the horizontal plane with vertical stimulus movements on the screen. Specifically, our participants used their mouse to respond to the font color of stimuli: They dragged each stimulus either to the lower or upper screen location. In sum, three experiments showed that this paradigm is appropriate for reliably replicating spatial congruency effects.

In the first two experiments, we examined language-space associations of nouns referring to entities typically located in a lower or an upper vertical location (see Lachmair et al., 2011). We used English (Experiment 1) and German (Experiment 2) materials and recruited English (Experiment 1) and German (Experiment 2) native speakers. In both experiments, response times (i.e., the time period from the appearance of the noun on the computer screen until initiating the mouse movement) were faster when the referent’s typical vertical location matched the dragging direction. This corresponds to the spatial congruency effect obtained with the lab-based version of the manual vertical Stroop paradigm (Lachmair et al., 2011, Experiment 2). Consequently, the experiments provided first empirical support in favor of our web-suited paradigm. In addition, the results revealed that our paradigm seems to work in different languages and populations.

In Experiment 3, we aimed at assessing the effectiveness of the paradigm with respect to further vertical spatial associations. To this end, we investigated valence words referring to emotions that are associated with a slouched or an upright bodily posture (i.e., with spatial experiences that are characterized by having a down vs. an up component). Interestingly, the investigation of such valence-space associations has become of ever-increasing interest since the study of Meier and Robinson (2004) was published. Crucially, we again observed a spatial congruency effect (faster responses when the vertical association of the words matched the dragging direction) that was in line with previous lab-based work using the manual vertical Stroop task (see Dudschig et al., 2015, Experiment 3). This clearly indicates that the applicability of our paradigm is not restricted to investigating a specific type of language-space association. Rather the paradigm seems to constitute a reliable web-suited approach to examining a variety of issues related to vertical spatial associations.

Moreover, the current results indicate that the emergence of spatial congruency effects stemming from language-space associations (i.e., faster responses when the vertical association of the word matches the response location; Dudschig et al., 2015, Experiment 3; Lachmair et al., 2011, Experiment 2) does not necessarily require actual vertical response movements. Whereas participants perform vertical arm movements to respond to the font color of words in standard lab-based paradigms, our participants moved their mouse on the horizontal plane to drag the words to the lower or upper screen location. Nevertheless, implementing a close relationship of manual actions (i.e., the hand movement when operating the mouse) and sensible action effects (i.e., seeing the word moving downwards or upwards, respectively) appears to be an essential factor as visual feedback per se could not reliably provoke spatial congruency effects in setups with simple stationary keypress responses (Schütt et al., 2021).

The exact origin of the spatial congruency effects obtained by applying the mouse-based paradigm remains vague. We suggest two possible mechanisms. Firstly, the congruency effects could have originated from the correspondence of spatial information conveyed by the linguistic stimuli (e.g., “worm” is related to a lower vertical location) and planning a mouse movement associated with visual action effects (i.e., a word movement) towards the lower or upper end of the screen. This approach is clearly in line with the idea of ideomotor theory, according to which representing anticipations of action effects is crucial to action planning (for overviews, see Badets et al., 2016; Hommel et al., 2001; Shin et al., 2010). Of course, it may be argued that the participants in lab-based paradigms were also exposed to visual action effects as they should have seen their arms moving upwards and downwards. For the present study, however, we can rule out the possibility that actual upward and downward arm movements caused the observed spatial congruency effects. Secondly, it could be that participants—despite conducting forward and backward mouse movements on the horizontal plane—internally re-coded their responses as “up” and “down” movements (for related suggestions see, for example, Brass et al., 2003; Eder & Rothermund, 2008). Thus, the congruency effects would result from the correspondence of the spatial information conveyed by the linguistic stimuli and an internal verbal response code. This interpretation is also relevant and even more important in the context of prior lab-based research using vertical responses movements as participants could have assigned verbal codes (“up” vs. “down”) to their responses without the need of any re-coding.

In contrast to previous lab-based studies using vertical response devices with four keys (e.g., Lachmair et al., 2011), we observed spatial congruency effects not only for response times, but also for movement times. This might be due to several reasons. For instance, when operating vertical response devices with four keys (see Fig. 1), participants started trials by holding both middle keys, with each hand pressing one of the keys. They were then required to complete their response decision prior to releasing the appropriate middle key and initiating the vertical arm movement, as releasing the wrong middle key caused an error feedback. In the current studies, however, participants controlled the mouse with a single hand, which made it possible to activate the response hand before finishing the decision on the response direction. Interestingly, Vogt et al. (2019) made use of a vertical response device with a single middle key (see Fig. 1) when investigating language-space associations in children. Crucially, they also obtained a congruency effect on movement times, indicating that the described changes regarding the setup may indeed have an impact on movement times. In addition, requirements of the mouse-based paradigm with respect to response execution should be considered. For example, mouse movements could have been conducted less ballistically than response movements on vertical response devices used in lab-based setups, thus possibly being more sensitive to a correspondence of linguistic stimuli and response direction.

In the current research, we focused on collecting and evaluating data on response times, movement times, and response correctness to match our dependent variables with those measures that have typically been considered in prior lab-based research using vertical response movements to investigate language-space associations (e.g., Dudschig et al., 2015; Lachmair et al., 2011; Thornton et al., 2013). Potential future research applying our mouse-based paradigm could have a systematic look at the vast range of other metrics that have been tried and tested in the context of mouse-tracking designs, such as entropy (an indicator for movement complexity) or trajectory curvature (for a thorough overview see Wirth et al., 2020). In particular, exploiting temporally continuous real-time measures may help to gain insights into the dynamics of the investigated spatial congruency effects.

Importantly, our web-suited paradigm will be of interest to a wide range of research areas. For instance, *Conceptual Metaphor Theory* (e.g., Lakoff & Johnson, 1980, 1999) proposes that abstract concepts (e.g., “freedom, “democracy”, “justice”, and “love”) are mentally represented by a mapping onto concrete domains that can be experienced physically. In this context, it has frequently been suggested that abstract concepts are mapped onto the spatial domain. Especially vertical space plays a crucial role for grounding concepts such as morality (e.g., Hill & Lapsley, 2009; Zhai et al., 2018), divinity (e.g., Chasteen et al., 2010; Meier et al., 2007), power (e.g., Jiang & Henley, 2012; Schubert, 2005; Wu et al., 2016; Zanolie et al., 2012), and valence (e.g., Ansorge et al., 2013; Meier & Robinson, 2004; Santiago et al., 2012). Of course, our paradigm could also easily be modified to investigate horizontal spatial associations. For example, such spatial associations are of great importance in the research on the mental representation of time (e.g., Santiago et al., 2007; Ulrich & Maienborn, 2010; Weger & Pratt, 2008). Moreover, horizontal spatial associations play a significant role in the research area of *body-specificity*, which deals with disentangling cultural, linguistic, and bodily influences with respect to the grounding of abstract concepts (e.g., Casasanto, 2009; Casasanto & Henetz, 2012; Casasanto & Jasmin, 2010; de la Fuente et al., 2015; de la Vega et al., 2012; Li & Cao, 2019). Likewise, research on the spatial numerical association of response code (SNARC) effect is inherently based on the idea of spatial grounding (see Fischer & Shaki, 2014, for a recent review). Our paradigm might be particularly useful for the web-based investigation of the vertical SNARC effect (e.g., Gevers et al., 2006; Ito & Hatta, 2004; Schwarz & Keus, 2004).

In sum, the mouse-based paradigm establishes a reliable setup for replacing vertical response movements that typically require special response devices. The method is easy to implement, allows web-based data collection, and may also facilitate future lab-based research. Of course, the paradigm can easily be adapted for investigating associations in other spatial dimensions. It thus has the potential to be a valuable tool for research across a wide range of domains interested in spatial associations.

## Data Availability

All data sets are publicly available at https://doi.org/10.5281/zenodo.4557024. All materials are mentioned and described in the Method sections.

## Experimental stimuli used in Experiments 1 to 3

**Table:**

**Experiment 1: English location words**
abyss; canyon; carpet; cellar; clover; depth; ditch; diver; earth; floor; foot; grass; grave; ground; hell; mole; mouse; rails; river; root; sidewalk; soil; sole; stone; street; subfont; submarine; subway; swamp; tunnel; underworld; worm.
alps; balloon; bird; castle; ceiling; cloud; comet; crown; eagle; gable; gallery; hawk; height; highlands; hill; kite; maximum; moon; mountains; nest; plane; planet; roof; satellite; sky; skyscraper; star; summit; sun; top; tower; universe.
**Experiment 2: German location words**
Abgrund; Schlucht; Teppich; Keller; Klee; Tiefe; Graben; Taucher; Erdreich; Fußboden; Fuß; Gras; Grab; Boden; Hölle; Maulwurf; Maus; Schienen; Fluss; Wurzel; Gehweg; Erde; Sohle; Stein; Straße; Untergrund; U-Boot; U-Bahn; Sumpf; Tunnel; Unterwelt; Wurm.
Alpen; Ballon; Vogel; Burg; Decke; Wolke; Komet; Krone; Adler; Giebel; Empore; Falke; Höhe; Hochland; Berg; Drachen; Höhepunkt; Mond; Gebirge; Nest; Flugzeug; Planet; Dach; Satellit; Himmel; Hochhaus; Stern; Gipfel; Sonne; Spitze; Turm; Weltall.
**Experiment 3: German posture-specific emotional valence words**
aufgeregt (excited); begeistert (enthusiastic); freudvoll (joyful); gutgelaunt (good-humored); stolz (proud); freudig (joyous); ekstatisch (ecstatic); erheitert (exhilarated); erfreut (pleased); erleichtert (relieved); ermutigt (encouraged); optimistisch (optimistic); beschwingt (elated); glücklich (happy); jubelnd (jubilant); strahlend (radiant); fröhlich (cheerful); vital (lively); triumphierend (triumphant); vergnügt (jolly).
traurig (sad); bedrückt (glum); demütig (humble); bekümmert (concerned); betrübt (saddened); deppresiv (depressive); erschöpft (exhausted); elend (miserable); gebrochen (broken); entmutigt (discouraged); pessimistisch (pessimistic); leidvoll (sorrowful); trübsinnig (blue); schwermütig (melancholy); enttäuscht (disappointed); verzweifelt (desperate); trauernd (grieving); ohnmächtig (helpless); deprimiert (depressed); trostlos (dismal).

English and German location words were derived from the work of Lachmair et al. (2011) and are counterparts of each other (see the Method sections). Posture-specific emotional valence words were taken from Dudschig et al. (2015); English translations are given in parentheses

## Footnotes

1 Of course, a mouse-based paradigm also has the potential to replace specific response devices requiring vertical actions in future lab-based research on spatial associations.

2 The corpus that we used for determining the word frequency classes did not give a value for the noun “subfont”. Thus, we discarded this noun from the analysis of frequencies, which reduced the number of degrees of freedom in the *t*-test.

3 Simonsohn (2015) recommended for replications to have 2.5 times as many observations as the original study. This would yield a sample size of 45 participants. However, due to balancing demands, we needed a sample size that is a multiple of six. Hence, we set the target sample size to 48 participants.

## Code availability

The original and an updated version of the custom-made jsPsych plugin for implementing the mouse movement task can be found at https://doi.org/10.5281/zenodo.4557024. Analysis scripts will be uploaded at the same place upon publication.
